# Optimal B-spline Mapping of Flow Imaging Data for Imposing Patient-specific Velocity Profiles in Computational Hemodynamics

**DOI:** 10.1109/TBME.2018.2880606

**Published:** 2018-12-11

**Authors:** Alberto Gomez, Marija Marčan, Christopher J. Arthurs, Robert Wright, Pouya Youssefi, Marjan Jahangiri, C. Alberto Figueroa

**Affiliations:** Department of Biomedical Engineering, King’s College London, UK; Department of Biomedical Engineering, King’s College London, UK; Department of Biomedical Engineering, King’s College London, UK; Department of Biomedical Engineering, King’s College London, UK; Department of Cardiothoracic Surgery & Cardiology, St. George’s Hospital, London, UK; Department of Cardiothoracic Surgery & Cardiology, St. George’s Hospital, London, UK; Department of Biomedical Engineering, King’s College London, UK, Departments of Surgery and Biomedical Engineering, University of Michigan, Ann Arbor, MI, USA.

**Keywords:** CFD, Patient-specific Modelling, Flow Profile, Magnetic Resonance Imaging, Doppler Ultrasound

## Abstract

**Objective:**

We propose a novel method to obtainmap patient-specific blood velocity profiles (obtained from imaging data such as 2D flow MRI or 3D colour Doppler ultrasound) and map them to geometric vascular models suitable to perform CFD simulations of haemodynamics. We describe the implementation and utilisation of the method within an open-source computational hemodynamics simulation software (CRIMSON).

**Methods:**

tThe proposed method establishes point-wise correspondences between the contour of a fixed geometric model and time-varying contours containing the velocity image data, from which a continuous, smooth and cyclic deformation field is calculated. Our methodology is validated using synthetic data, and demonstrated using two different in-vivo aortic velocity datasets: a healthy subject with normal tricuspid valve and a patient with bicuspid aortic valve.

**Results:**

We compare the performance of our method with results obtained with the state-of-the-art Schwarz-Christoffel method, in terms of preservation of velocities and execution time. Our method is as accurate as the Schwarz-Christoffel method, while being over 8 times faster. The proposed method can preserve either the flow rate or the velocity field through the surface, and can cope with inconsistencies in motion and contour shape.

**Conclusions:**

Our results show that the method is as accurate as the Schwarz-Christoffel method in terms of maintaining the velocity distributions, while being more computationally efficient.Our mapping method can accurately preserve either the flow rate or the velocity field through the surface, and can cope with inconsistencies in motion and contour shape.

**Significance:**

The proposed method and its integration into the CRIMSON software enable a streamlined approach towards incorporating more patient-specific data in blood flow simulations.

## I. Introduction

PATIENT-specific computational fluid dynamics (CFD) enable a high-resolution, non-invasive description of space and time-resolved blood flow [1]. CFD models can be constructed from relatively few measurements of blood velocity, anatomy and pressure [2]. Typically, the patient’s vascular anatomy is obtained by segmenting 3D computed tomography (CT) or magnetic resonance (MR) image data. Performing accurate anatomical segmentations has always been recognised as a key piece in the puzzle of patient-specific modelling. Significant efforts have been made to produce robust segmentation algorithms to capture the complexity of vascular structures [3], [4]. However, not nearly enough attention has been devoted to the task of incorporating patient-specific velocity data into the simulation pipeline. With few exceptions, the standard approach has been to obtain a volumetric flow waveform from the velocity data, and then to impose an idealised velocity profile (e.g., plug, parabolic, Womersley) [5] at the corresponding geometric model face. It is however well-known that the impact of idealised inflow velocity profiles in CFD simulations is large [6]–[8], particularly in the ascending thoracic aorta, where the flow is highly dynamic and displays complex patterns [9]–[12]. The complexity increases in pathological conditions such as aortic valve disease and artificial and bio-prosthetic valves [13]. Of particular interest is Bicuspid Aortic Valve (BAV), the commonest congenital cardiac defect, with a prevalence of 1–2%. Its morbidity and mortality amount to more than that of all other congenital cardiac conditions combined [14]. It is commonly associated with aneurysms of the thoracic aorta [15], and the hemodynamic link between BAV morphology and aneurysm formation is the current topic of intense research.

In this paper we propose a new method to calculate patient-specific, time-resolved velocity profiles from image data (2D flow MRI and 3D colour Doppler) that optimally fit a fixed geometric model obtained from a single anatomical image (CT or MRI). We use a novel scheme which allows mapping a flat face of the geometric model to a segmented velocity image, which allows to incorporate the velocities from the image into the model. The main novelties of this paper are twofold: (1) formulation of an optimal B-spline mapping where the user can choose between maintaining flow rate or velocity distribution, and (2) implementation of the method into the CRIMSON (CardiovasculaR Integrated Modelling and SimulatiON) platform [16], an open-source blood flow simulation software which enables accessibility of the proposed method to the wider community.

This paper is organised as follows: related work on blood velocity measurements for patient-specific hemodynamic modelling is discussed in Sec. II. Section III describes the technical details of the method: obtaining a velocity profile from velocity data and mapping a fixed geometric model to the velocity profile (Sec. III-A), cyclic interpolation of the profiles over the cardiac cycle (Sec. III-B), controlling the trade-off between velocity and flow (Sec. III-C), and method implementation in CRIMSON [16] (Appendix A). Sec. IV describes the synthetic and in-vivo data. Section V describes the results. Lastly, sections VI, VII and VIII provide a critical discussion of the results, method limitations, and conclusions.

## II. Related Work

The most widespread technique for measuring blood velocity in the clinic is Doppler ultrasound [17], [18]. Pulsed Wave Doppler (PWD) ultrasound allows measuring the component of blood velocity parallel to the sound direction over time at a given location. Doppler measurements must therefore be angle-compensated [19]. To use PWD to prescribe boundary conditions in CFD, one must assume an idealised velocity profile which is adjusted to match the mean or maximum velocity. AlternativelyIf available, 3D Colour Doppler Imaging (CDI) can be used to obtain velocity over the entire cross section of a vessel [20], [21], allowing for specification of patient-specific velocity profiles. Velocity measurements over the vessel cross section can also be obtained with 2D flow MRI [22]. Hardman et al. [6] compared CFD results obtained using an idealised profile (defined by centre-line velocity data from flow MRI), with i) a profile defined by single through-plane velocity components, and ii) a profile defined by a three-component velocity data. Their study suggests that while use of three-component velocity does not have a major influence in the CFD results (except for capturing finer details in the flow helicity), using the through-plane component of the velocity significantly affects the simulation results compared to those obtained using an idealised profile. Chandra et al. [23] also concluded that the use of 3-component velocity data has little impact on the simulation results compared to 1-component data. Similar findings appeared in [8], for healthy subjects. It should be noted, though, that a more recent study [12] on patients with abnormal aortic valve suggested that neglecting in-plane velocities at the inlet yield underestimated average and maximum velocities in the ascending aorta. Youssefi et al. [13] used through-plane patient-specific velocity profiles to assess differences in flow asymmetry and wall shear stress in patients with an array of valvular pathologies, finding significant differences compared to healthy volunteers for whom the aortic inflow velocity can be reasonably approximated by a parabolic profile.

A key problem to incorporate patient-specific velocity profiles in CFD simulations is the spatial mapping between the (generally fixed) geometric model inlet or outlet face and the time-varying velocity data. The geometric data and the velocity images may be acquired at different times and even using different techniques (e.g., CT-derived anatomy and MRI velocity data). The vessel motion (bulk and pulsatile changes in cross section) recorded in the velocity data is generally not incorporated into the CFD model, which often assumes the vessels to be rigid [5], [6], [8], [23]–[26]. Only when anatomical and velocity data come from the same source, and the CFD model accounts for a moving wall (e.g. a fluid-structure interaction simulation [1]), the mapping between velocity and geometric model might not be needed. Typical modelling approaches have assumed that the spatial mapping between geometric model and velocity data is not necessary because the deformations of the vessel of interest are small [5], [24], [27], e.g. at the carotid arteries.

Leuprecht et al. [28] proposed a surface fitting of the velocity measurements limited to the inlet cross section of the geometric model. This method requires fine-tuning of the fitting parameters to avoid non-zero velocity values at the contour. A simpler approach was proposed by Hardman et al. [6], who used a mapping limited to a rigid alignment of the centroids of the geometric model inlet contour (obtained from CT) and the velocity data (obtained from flow MRI). This approach was insufficient because in addition to a bulk motion during the cardiac cycle, some vessels experience significant changes in cross-sectional area. The ascending thoracic aorta is a prime example of this behaviour.

More recentlyPrevious work, [23], [25] computed the deformation between the inlet face of the geometric model and the velocity images (flow MR) using the Schwarz-Christoffel (SC) method. This method maps the surface of a closed polygon to a unit circle [29]. Thus, building a map between the geometric model and the velocity data requires two SC mappings: one from the geometric model to the unit circle, and a second from the unit circle to the velocity data. The SC method may have convergence problems for large number of nodes [29] which could prevent the adequate mapping of some contours. The SC methodMoreover, it requires a point-wise correspondence between the geometric model contour and the segmented contour in the velocity data, and the mapping depends on the centre location (not always obvious in abnormal valve geometries). These two contours will generally be defined in different coordinate systems, hence point-wise correspondence cannot be ensured. To the best of our knowledge, this potential inconsistency between coordinate systems of anatomy and velocity image data is obviated in SC-based published work.To the best of our knowledge, SC-based published work assumes that both contours are centred and rotationally aligned, however this is only true if anatomical and velocity data were acquired with the same imaging modality, during the same procedure, and without patient motion in between acquisitions. This is in general not true.

Another limitation of previous work is that mapping was carried out frame-by-frame. Therefore, the temporal smoothness and cyclic behaviour of the mappings is neglected, potentially affecting the numerical stability of flow simulations. Because the (fixed) surface area of the geometric model generally differs from that of the time-varying contours of the velocity data, a correction is required in the mapping to ensure preservation of flow rate. Previous work [23], [25], [28] maintained flow rate by scaling the velocities with the ratio between the surface areas of the velocity contours and the geometric model contour.

Another major difficulty in incorporating patient-specific inflow data into CFD simulation workflows is that there is currently no publicly available software capable of performing mappings between anatomical and velocity data. Previous studies [5], [6], [23]–[25], [27], [28] used ad-hoc implemenations, limiting accessibility from the community.

In this paper, we developed a novel velocity mapping method capable of handling large deformations and motions and implemented it in CRIMSON [16], a publicly available hemodynamic simulation package.

## III. Methods

**Fig. 1:**
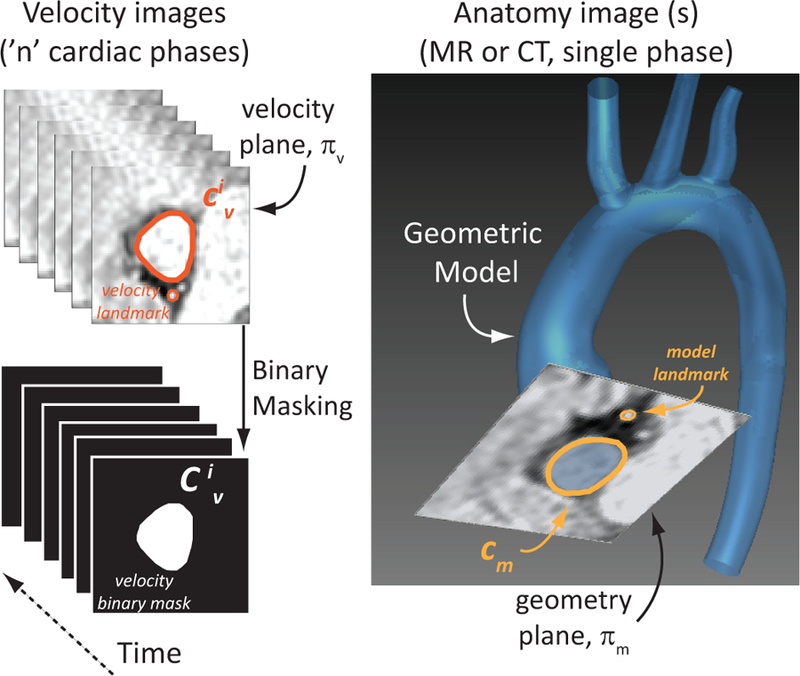
Method overview. Through-plane velocity is extracted from 3D colour Doppler or 2D flow MRI data (left). For each temporal phase *i* in the velocity data, a velocity contour $c_{\upsilon }^{i}\subset \pi_{\upsilon }$ defining the boundary of the vessel is obtained (left). A separate model contour $c_{m}\subset \pi_{m}$ is defined in the face of the geometric model, built from the anatomy image data (CT or MRI; right). In this work, $c_{m}$ is not time-dependent, but this need not be the case in general. In order to co-register $c_{\upsilon }^{i}$ and $c_{m}$, the user must define landmarks in both velocity and anatomy images. The velocity profiles between each of the *n* cardiac phases are temporally interpolated to produce the required resolution for the CFD analysis

The proposed method is summarised in Fig. 1 and detailed in Sec. III-A to III-C. Implementation details in CRIMSON are described in Appendix A. Briefly, blood velocity data (2D flow MRI or 3D colour Doppler) is acquired at the location of interest. For each cardiac phase in the velocity image sequence (typically, a few dozen), the lumen is segmented and the dense deformation between the lumen contour in the velocity data and the corresponding contour in the geometric model is calculated. The trade-off between maintaining flow rate or velocity in the mapping process must be specified by the user. Finally, a smooth cyclic temporal interpolation is obtained to produce velocity data for the CFD model: typically, thousands of time points in one cardiac cycle.

### A. Mapping Geometric Model to Velocity Images

The method presented here only considers the through-plane component of the velocity, however it could be easily generalized to a three-component velocity scenario. Let $c\subset {\mathbb{R}}^{3}$ be a closed, non-self-intersecting planar curve contained in a plane Π_*c*_. Denote the set of all such curves by

$$\chi :=\left\{c{|c\subset \prod_{c}\subset {\mathbb{R}}^{3},\text{for}\;\text{some}\prod_{c}≅\mathbb{R}}^{2}\right\}.$$

For each cardiac phase *i* = 1, …, *n*, a velocity contour $c_{\upsilon }^{i}\in \chi$ delineating the vessel wall in the velocity image data must be produced, together with an associated binary mask $C_{\upsilon }^{i}:\prod_{c}\equiv \prod_{\upsilon }\to \left\{0,1\right\}$, where $\prod_{\upsilon }$ is the plane containing the velocity image data, such that $C_{\upsilon }^{i}$ takes the value 1 inside $c_{\upsilon }^{i}$ and 0 outside it. Similarly, a corresponding contour on the anatomy image, $c_{m}\in \chi$ must be obtained on $\prod_{m}$, the plane containing the face of the geometric model which will be mapped to the velocity data. In this work, *c*_*m*_ is fixed in time, but this need not be the case in general. In practice, *c*_*m*_ is either a polygonal if the geometric model is given by a surface triangulation (e.g., .stl file) or an analytical curve in the case of a CAD model. There are a wide variety of tools available for image segmentation [4]. In this paper, we used CRIMSON’s [16] semi-automatic segmentation toolbox.

The contours $c_{\upsilon }^{i}$ and $c_{m}$ will generally be in different coordinate systems and have slightly different shapes. In this paper, we perform a rigid alignment followed by a non-rigid mapping between $\prod_{\upsilon }$ and $\prod_{m}$, restricted to points inside $c_{\upsilon }^{i}$ and *c*_*m*_, respectively.

#### 1) Rigid alignment of $c_{\upsilon }^{i}$ and $c_{m}$:

The rigid mapping is expressed as a matrix transformation. Here, we work in a subset of real projective space $ℍ:=\left\{\left(x,y,z,w\right)\in {\mathbb{P}}^{3}|w=1\right\}≅{\mathbb{R}}^{3}$; $ℍ\;\text{is}\;{\mathbb{P}}^{3}$ without the point at infinity, and provides a system of homogeneous coordinates. In what follows, let j∈{υ,m}. For each contour on the velocity and anatomy images, consider the associated plane $\prod_{j}$. Let *B*_*j*_ be the orthonormal bases with third component given by the unit normal to the associated plane, chosen to be pointing in the same direction relative to the anatomy in both $B_{\upsilon }$ and $B_{m}$, neglecting the *w*-component so that these have only *x*, *y* and *z* entries. Define the change of basis matrices

$$M_{j}=\left[\begin{array}{cccc} \vdots & \vdots & \vdots & 0\\ B_{j}^{1} & B_{j}^{2} & B_{j}^{3} & 0\\ \vdots & \vdots & \vdots & 0\\ 0 & 0 & 0 & 1 \end{array}\right]$$

where the $B_{j}^{k}\in B_{j},k\in \left\{1,2,3\right\}$, are column vectors. $M_{j}^{-1}\prod_{j}$ is then contained in a plane with z≡z(j),$\prod_{z\left(j\right)}$. Applying these transformations thus maps *c*_*m*_ and *c*_*v*_ into parallel planes such that the contours can then be mapped into the same plane and simultaneously aligned with one another by applying a translation which is computed as follows: Consider a set of points $P_{j}:=\left\{p_{j}|p_{j}\in c_{j}\right\}$, given in homogeneous coordinates. Note that due to the previous transformation,$M_{j}^{-1}P_{j}\|\prod_{xy}$.*P*_*j*_ may consist of vertices of a polygonal curve, or uniformly distributed points on an analytic curve. The centroids of the $M_{j}^{-1}P_{j}$ are given by

$$O_{j}:=\frac{1}{\left|P_{j}\right|}\sum_{P_{j}}M_{j}^{-1}P_{j}.$$

then,

(1) $$T_{j}=\left[\begin{array}{cccc} 1 & 0 & 0 & O_{j,x}\\ 0 & 1 & 0 & O_{j,y}\\ 0 & 0 & 1 & O_{j,z}\\ 0 & 0 & 0 & O_{j,w} \end{array}\right]$$

defines translation by $O_{j}$; note that $O_{j,w}\equiv 1$. Thus,

$$P_{j}^{2D}:=T_{j}^{-1}M_{j}^{-1}P_{j}\in \prod_{xy}$$

gives the set of points on each contour mapped into $\prod_{xy}$ with centroids collocated at the origin.

The contour points $P_{m}^{2D}$ must now be rotated about their centroids to complete the rigid alignment with $P_{\upsilon }^{2D}$ The user identifies a single anatomical landmark in both the Π_*j*_; call the landmark’s location in each plane *L*_*j*_. Let $\theta_{j}$ be the angle between the *x*-axis and $T_{j}^{-1}M_{j}^{-1}L_{j}$ in $\prod_{xy}$ (with the anti-clockwise direction taken to be positive), then define a rotation matrix

$$R_{j}=\left[\begin{array}{cccc} cos\left(\theta_{j}\right) & -sin\left(\theta_{j}\right) & 0 & 0\\ sin\left(\theta_{j}\right) & cos\left(\theta_{j}\right) & 0 & 0\\ 0 & 0 & 1 & 0\\ 0 & 0 & 0 & 1 \end{array}\right]$$

**Fig. 2:**
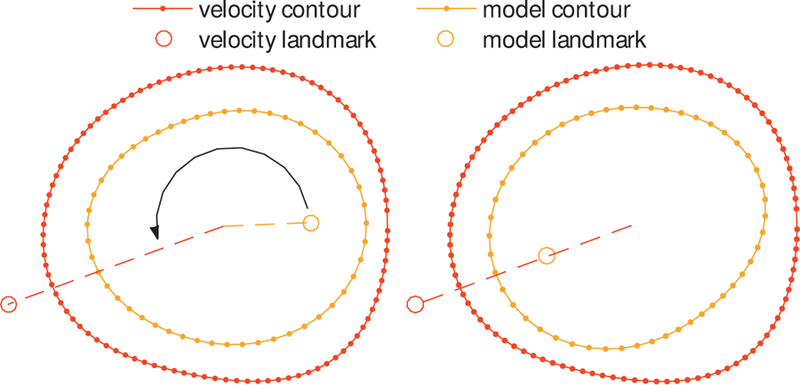
Rotational contour alignment using a reference landmark. In what follows, the difference in shape and size between the $c_{m}$ and $c_{\upsilon }^{i}$ the figures in this section is exaggerated for ease of visualisation.

The final aligned contour points are now given by

$$P_{m}^{aligned}:=R_{\upsilon }R_{m}^{-1}T_{m}^{-1}M_{m}^{-1}P_{m}$$

and

$$P_{\upsilon }^{aligned}:=T_{\upsilon }^{-1}M_{\upsilon }^{-1}P_{\upsilon }.$$

which describe the aligned contours $c_{\upsilon }^{aligned}$ and $c_{m}^{aligned}$. The effect of this final transformation, $R_{\upsilon }R_{m}^{-1}$, is shown in Fig. 2, where the velocity contour points, $T_{m}^{-1}M_{m}^{-1}P_{m}$ are rotated to achieve rigid alignment with the model contour points (right). Note that previous work assumes that this alignment is given but this is generally not the case. The next step is to apply a smooth deformation field to match the contours shape.

The matrix *R* can then be applied to the points in the model contour shown in Fig. 2 (left) to yield the rigidly aligned model contour shown in Fig. 2 (right). Note that previous work assumes that this alignment is given but this is generally not the case. The next step is to apply a smooth deformation field to match the contours shape.

#### 2) Non-rigid Mapping of the Model Contour to the Imaging Contour:

Related literature discussed in Sec. II utilizes the Schwarz-Christoffel (SC) mapping for non rigid mapping of the rigidly aligned contours. In this paper we propose using a uniform B-spline vector field that deforms and interpolates the interior of the flat inlet face of the geometric model to the velocity image data, which enables sampling of the velocity imaging data at the locations required by the geometric model. Uniform B-spline vector fields are continuous, smooth piecewise functions defined on a uniform grid of control points, widely used in computational imaging and signal processing for providing computational efficiency [30] and control over the smoothness of the deformation.

**Fig. 3:**
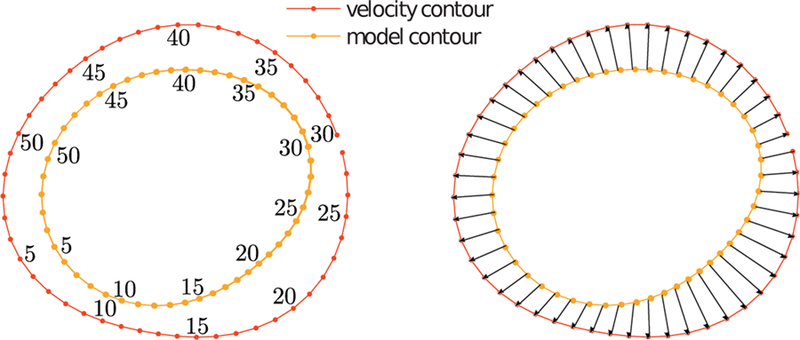
Non rigid alignment between the velocity image derived contour and the model contour at the inlet. Left: point-wise correspondence between points defining equal angular increments in the two contours (only every fifth point is labelled). Right: the corresponding points specify the contour deformation vectors that will define the mapping.

In order to establish correspondences between the two rigidly aligned contours, we first specify an initial point-wise correspondence between the two. The SC method needs that the contour is in the form of a polygon and requires a non-trivial computation of the pre-vertices [29]. In our case, we proceed as follows. We first compute the analytical aligned contours $c_{j}^{aligned}$ by fitting a smooth closed spline on the vertices $P_{j}^{aligned}$. Then we define $Q_{j}^{aligned}=\left\{q_{j}\left(2\pi i/K\right)|q_{j}\left(2\pi i/K\right)\in c_{j}^{aligned},i=1,\ldots ,K\right\}$, evenly distributed between 0 and 2π on $c_{j}^{aligned}$ as shown in Fig. 3 (left). This permits us to establish corresponding points, and also to handle different number of vertices on the original contours. Conveniently, this approach also allows us to use non-polygonal shapes, e.g. analytical contours, if available. The corresponding points determine *K* vectors

(2) $$V:=\left\{v:=q_{\upsilon }\left(2\pi i/K\right)-q_{m}\left(2\pi i/K\right)|q_{j}\left(2\pi i/K\right)\in Q_{j}^{aligned},j\in \left\{\upsilon ,m\right\},i=1,\ldots ,K\right\},$$

as shown in Fig. 3 (right) for *K* = 50.

**Fig. 4:**
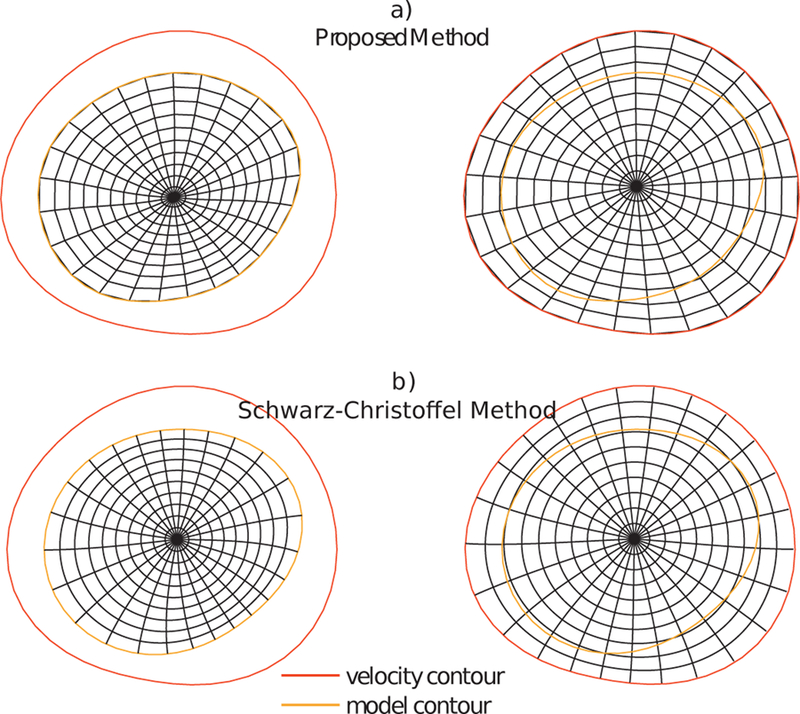
Mapping from model inlet to velocity profile, using the proposed method (top) and the Schwarz-Christoffel method (bottom). The difference in shape between the two contours has been exaggerated for better visualisation of the smooth transition between contours offered by the proposed method.

The non-rigid mapping $\prod_{m}\to \prod_{\upsilon }$ between the aligned contours is computed by minimizing the fitting error e(**f**):

(3) $$e\left(\text{f}\right)=\sum_{k=1}^{K}{\left\|\text{f}\left(Q_{m}^{aligned}\right)-\text{v}\right\|}^{2}$$

for $\text{f}\;\text{=}\;\left[f_{x}f_{y}\right]$ being a dense, smooth vector field. We propose to solve this minimization problem by representing the mapping **f** in a B-spline basis:

(4) $$f_{x}\left(q_{m}^{aligned}\right)=\sum_{i,j}c_{i,j}^{x}\beta \left(q_{m,x}^{aligned}/a-i\right)\beta \left(q_{m,y}^{aligned}/b-j\right)\;f_{y}\left(q_{m}^{aligned}\right)=\sum_{i,j}c_{i,j}^{y}\beta \left(q_{m,x}^{aligned}/a-i\right)\beta \left(q_{m,y}^{aligned}/b-j\right)$$

where *β* is the cubic B-spline piecewise basis function, [*a b*] is the separation between control points in the B-spline control grid, and ${\left\{c^{x},c^{y}\right\}}_{i,j}$ are the B-spline weights for the *x* and *y* components of the resulting field at each control point [30]. Equation (4) can be evaluated at the corresponding points and expressed as a matrix product:

(5) v = Bc

where **v** is a matrix where each row is a correspondence vector from Fig. 3, *B* is a matrix with the B-spline bicubic tensor product evaluated at each corresponding point, for each B-spline control point; and **c** is a matrix where each row is a tuple $\left[c^{x}c^{y}\right]\in {\mathbb{R}}^{2}$ for each B-spline control point. Details on B-spline fitting in general and on how to construct the above matrices particularly for vector problems can be found in [30], [31]. The goal is to find the coefficients **c** that verify (5). There is, in general, no exact solution for this problem; instead, we search for the *N* B-spline coefficients **c** that minimize the cost function $J:{\left({\mathbb{R}}^{2}\right)}^{N}\to \mathbb{R}$ derived from (3):

(6) $$J\left(c\right)=\left(1-\mu \right){\left\|\text{v}-B\text{c}\right\|}^{2}+\mu G\left(\text{c}\right)$$

where μG(c) is a regularisation term, whose contribution is controlled by the value of the scalar *µ*. This term is particularly important in this case because the input data is sparsely distributed within the B-spline domain (i.e., input data points are concentrated along the contour of the inlet), and as a result regularisation will guarantee a smooth behaviour elsewhere. This also allows us to use a coarser B-spline grid to have a better fit of the correspondence vectors. In the experiments presented later, we empirically chose μ=0.1 and a B-spline grid spacing of half the diameter of the smallest contour. An example of the mapping resulting from this dense deformation is is shown in Fig. 4, compared to the SC mapping on the same geometry.

#### 3) Full Mapping: Model Inlet to Velocity Profile:

Given a point set *P*_*m*_ on the model face where the velocity field is to be imposed, the velocity value can be obtained by mapping *P*_*m*_ to its corresponding positions in the velocity image, *P*_*v*_, and interpolating the velocity value. Concatenating the transformations described in previous sections yields:

(7) $$P_{\upsilon }=M_{\upsilon }T_{\upsilon }\text{f}\left(R_{\upsilon }R_{m}^{-1}T_{m}^{-1}M_{m}^{-1}P_{m}\right)$$

The velocity values at the locations required on the model face sampled from the velocity imaging data can therefore be computed as

(8) $$\upsilon \left(P_{\upsilon }\right)=L_{\upsilon }\left(P_{\upsilon }\right)$$

where $L_{\upsilon }\left(x\right)$ is the conventional linear interpolation operator on the velocity image at location *x*. The proposed mapping has been formulated independently of the dimensionality of the velocity; if 3 components of the velocity are available from the imaging data (e.g., from 4D Flow MRI), the method holds and $L_{\upsilon }\left(x\right)$ is a tri-linear interpolator.

### B. Cyclic and Smooth Interpolation of the Resulting Temporal Velocity Profiles

This mapping process described above is carried out for each cardiac phase in the imaging data, as is done in related literature using the SC method. In general, the CFD pipeline requires that prescribed boundary conditions have high temporal resolution, which normally far exceeds that available from the imaging data. For example, typical image acquisition rates would be up to 30 phases per cycle in 2D Flow MRI and 20 phases per cycle in 3D CDI, while the modelling would require a temporal resolution beyond 1000 phases per cycle. In this paper, we propose to interpolate the mapped velocity profiles at the required modelling temporal resolution using interpolating cyclic B-splines, which interpolate the mapped velocity profiles (one for each input velocity phase) over time to the desired temporal resolution. In our current formulation, this process is separate from the frame-wise mapping and therefore could be applied to other frame-wise mapping methods, such as the SC method. Provided a cycle interval $t\in [t_{0},t_{1})$, the through-plane velocity value *v*(*t,* **x**) at the location **x** of the rigid model inlet is redefined from (4) as:

(9) $$\upsilon \left(x,y,t\right)=\sum_{i,j}c_{i,j,k}\beta \left(x/a-i\right)\beta \left(y/b-j\right)\;\;\;\;\;\;\;\;\beta \left(\left(\left[t-t_{0}\right]\;(\mathrm{mod}\;t_{1}\right)\right)/\Delta t-k)$$

which analogously to (5) can be expressed in matrix form as

(10) $$\upsilon \left(\text{x,}t\right)=B\left(\text{x,}t\right)c_{t}$$

The coefficients *c*_*t*_ can be found by minimising *J*_*t*_:

(11) $$J_{t}\left(c_{t}\right)={\left\|\upsilon \left(\text{x,}t\right)-B\left(\text{x,}t\right)c_{t}\right\|}^{2}$$

In this case regularisation is not normally needed since samples (i.e., velocity profile images) are uniformly distributed over time and the space between B-spline control points Δt can be chosen so that there are several (typically two or more) time samples between every two control points. Note that (11) is defined here as a 2D+t smoothing and interpolation problem, in which case spatial smoothing is also achieved. Alternatively, the temporal smoothing and interpolation problem can be formulated in 1D (time) for each point in the model inlet, without any spatial smoothing.

### C. Controlling the Trade-off between Velocity and Flow

In general, the model and the velocity image contours at the inlet have slightly different shape and surface area. This is due to: 1) differences in imaging modality and acquisition time between anatomical imaging data for building the geometric model and imaging data to measure velocity; 2) segmentation errors; and 3) the way motion and changes in cross section of the vessel are taken into account in the model and in the velocity data. For these reasons, although the velocity distribution and the average velocity are maintained throughout the mapping process, the surface area is not. As a result, in general there will be a difference in the flow rate between the boundary condition prescribed to the CFD and the velocity data.

Unfortunately, it is not possible to maintain both the flow rate and the velocity distribution if there is a change in area. In this paper we introduce a user-selected scalar trade-off factor, *λ* which determines whether the velocity distribution is maintained (λ=0), the flow rate is maintained (λ=1) or any intermediate scenario (0<λ<1). This is achieved at the interpolation step described in the previous section. The final velocity $\upsilon_{f}$ is a function of the interpolated velocity *v* and the model and velocity image surface areas, $A_{model}$ and $A_{image}$ respectively:

(12) $$\upsilon_{f}=\upsilon \left(\left(1-\lambda \right)+\lambda \frac{A_{model}}{A_{image}}\right)$$

If the velocity *v* is has 3 components (e.g. it was provided by 4D Flow MRI), all components are affected by the same scaling (otherwise, unrealistic flow trajectories would appear). This scaling might not be necessary when several phases are used for defining a time-varying geometric model inlet from image data, in the context of large deformation fluid-structure interaction simulations.

### D. Software Availability for the Community

The described method has been implemented and made freely available for download as part of the CRIMSON environment, as described in detail in Appendix A. The CRIMSON implementation additionally provides the option to perform spatial smoothing of the velocity profile before it is imposed as a boundary condition on a vascular model. This is achieved by using a mass-preserving Gaussian kernel (see Appendix B); the mass-preserving aspect is key, as it is important to avoid artificially changing the cardiac output implied by the imposed profiles.

## IV. Materials and Experiments

### A. Experiments on Synthetic Data

**Fig. 5:**
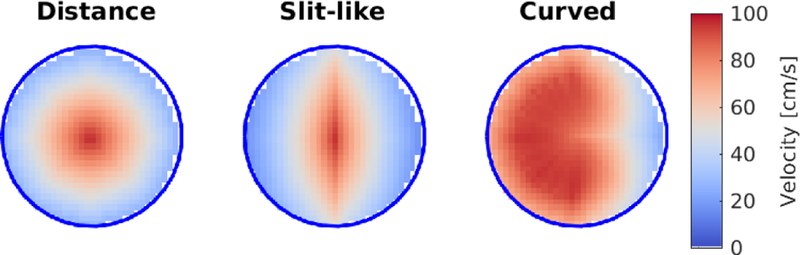
Test synthetic profile types, representing a variety of shapes that model simplified normal and abnormal aortic inlet velocity profiles.

We carried out experiments on synthetic data to assess the ability of the proposed method to map velocities between two different surfaces, and to compare it with the Schwarz-Christoffel (SC) mapping, which is used in related published work described in Sec. II. We used a MATLAB non-parallel implementation of our mapping method and the SC mapping MATLAB toolbox by Driscoll [37]. We produced *N* = 1000 pairs of inflow contours, using closed spline curves with 8 control points with random radii uniformly distributed in [1.05, 2.15] range, representative of those found in the human aorta [38]. To create closed polygons, the spline curves were sampled at 30 equally spaced locations. Rigid alignment (rotation and translation) was not considered for these experiments because related literature does not account for that. The area enclosed by contours corresponding to velocity imaging was uniformly sampled in a regular grid with a resolution of 0.1×0.1 mm, and for each pair of contours three profile types (shown in Fig. 5) were mapped: 1) Distance to edge profile (computed using a morphological distance operator on the regular grid), 2) Slit-like profile (anisotropic Gaussian masked by the first profile), and 3) Curved profile (curved Gaussian masked by the first profile). Velocity profiles were normalized to the range [0, 100] cm/s.

Quantitative evaluation was carried out on three measurements: 1) the difference in velocity distribution between the original velocity image and the mapped velocity; 2) The point-wise difference in mapped velocity between the SC method and the proposed method; and 3) the execution time for each mapping process. To compute the difference in velocity distributions between the original profile *A* and the mapped profile *B*, we computed the velocity histograms *h*_*A*_ and *h*_*B*_ and used the quadratic-chi (QC) histogram distance proposed in [39] to measure similarity between histograms in image analysis:

(13) $$QC_{m}^{A}\left(h_{A},h_{B}\right)=\sqrt{\sum_{ij}\left(\frac{h_{A,i}-h_{B,i}}{{\left(\sum_{c}\left(h_{A,c}+h_{B,c}\right)A_{c,i}\right)}^{m}}\right)\left(\frac{h_{A,j}-h_{B,j}}{{\left(\sum_{c}\left(h_{A,c}+h_{B,c}\right)A_{c,j}\right)}^{m}}\right)A_{i,j}}$$

where *m* = 0.5 and $A_{i,j}=1-\frac{\left|h_{A,i},h_{B,j}\right|}{\max_{i,j}\left|h_{A,i},h_{B,j}\right|}$ is a square matrix that measures the distances between all bins.

### B. Experiments on In-vivo Data

To demonstrate the practical applicability of the method, we will consider velocity and anatomical data corresponding to the ascending aorta of adult subjects: one healthy volunteer with normal tricuspid aortic valve and a second patient with pathological bicuspid aortic valve (BAV) and a diagnosis of severe aortic stenosis. The velocity data are prescribed on a plane at the sinotubular junction, and the aortic geometry is reconstructed from a single magnetic resonance angiography image and thus is assumed rigid throughout the cardiac cycle. Anatomical images to build the model were acquired using Magnetic Resonance Imaging (MRI) and velocity measurements were carried out using colour Doppler ultrasound on the volunteer and 2D flow MRI on the patient.

Anatomical MRI was carried out on both the volunteer and the patient using standard of care Cardiac MR to image the entire thoracic aorta, including the head and neck vessels using a Philips Achieva 3T scanner with a breath-held 3D fast gradient echo sequence. The patient underwent gadolinium-enhanced MR Angiography (0.3 ml/kg; gadodiamide, Omniscan, GE Healthcare). Slice thickness was 2.0 mm, with 56–60 sagittal slices per volume. A 344 × 344 acquisition matrix was used with FoV of 35*cm×*35 cm (reconstructed to 0.49×0.49×1.00*mm*). Other parameters included a repetition time (TR) of 3.9 ms, echo time (TE) of 1.4 ms, and a flip angle of 27°.

Doppler ultrasound images were acquired using a Philips iE33 system with a X3–1 transthoracic transducer, over 7 beats and maximising the Doppler range to avoid aliasing. Images were acquired from an apical window ensuring that the entire cross section of the aortic valve (AV) was within the FoV.

Time-resolved, velocity encoded 2D anatomic and through-plane Phase Contrast (PC)-MRI (2D flow MRI) was performed on a plane orthogonal to the ascending aorta at the sino-tubular junction. Imaging parameters included TR, TE, and flip angle of 4.2 ms, 2.4 ms, and 15°, respectively. The FoV was 35 × 30 cm with an acquisition matrix of 152 × 120, and a slice thickness of 10 mm, resulting in a voxel size of 2.3×2.4×10 mm (resampled at 1.37 × 1.36 × 10 mm). Data acquisition was carried out with a breath-hold and gated to the cardiac cycle. Velocity sensitivity was adjusted to avoid aliasing. Cine sequences at the level of the AV (5–8 slices) were performed for assessment of valve morphology.

Quantitative and qualitative experiments were carried out to assess the quality of the mapping, focusing on the aspects of the mapped velocity that may be of higher relevance for CFD simulations. Quantitatively, and similarly to our experiments on synthetic data, we measured the difference in velocity distribution after mapping. We also measured differences in flow rate and peak velocity for values of the trade-off factor λ∈{0,0.25,0.5,0.75,1}. Qualitatively, we show the resulting velocity profile through the mapping process on a few selected phases of the systolic part of the cardiac cycle for both subjects.

## V. Results

### A. Results on Synthetic Data

Table I shows the QC distance [39] between the original velocity distribution (histogram) over the contour defined in the velocity image and the distribution of the mapped velocities. Average distance using our method and the S-C method error are similar, and a t-test showed no statistical difference between them (*α* = 0.01).

**Fig. 6:**
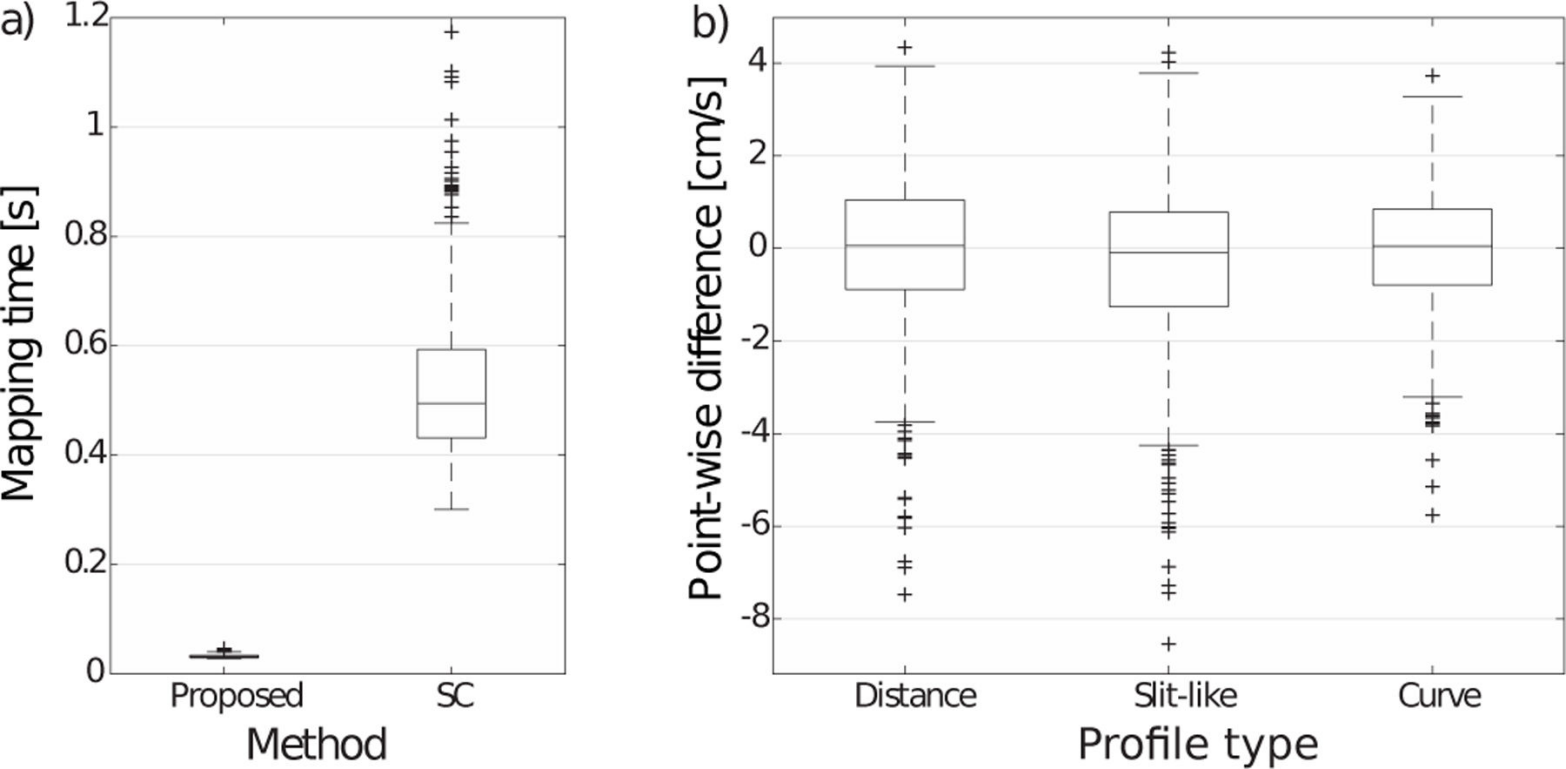
Quantitative analysis of profile mapping using synthetic data. (a) Execution time, per case, using the proposed method (left) compared to the SC method (right). (b) Point-wise difference in mapped velocity values between the proposed method and the SC method, using the three profile types from Fig. 5 (adapted to randomly generated contours). The error values in cm/s can also be read as % since the maximum velocity value was set to 100cm/s.

Fig. 6 a) shows the execution time (in s) for the mapping computation, using the proposed method (left) and the SC method (right). The proposed method was found to be over 8 times faster in average (*p <* 0.01).

Fig. 6 b) shows the point-wise difference between the two methods (in cm/s), for each profile type. The boxes show the median and the 25 and 75 quantiles of the absolute difference. The whiskers show the most extreme values not considered outliers, and outliers are shown with asterisks. The values for the three profiles were found to not be statistically different(*p <* 0.01).

**Fig. 7:**
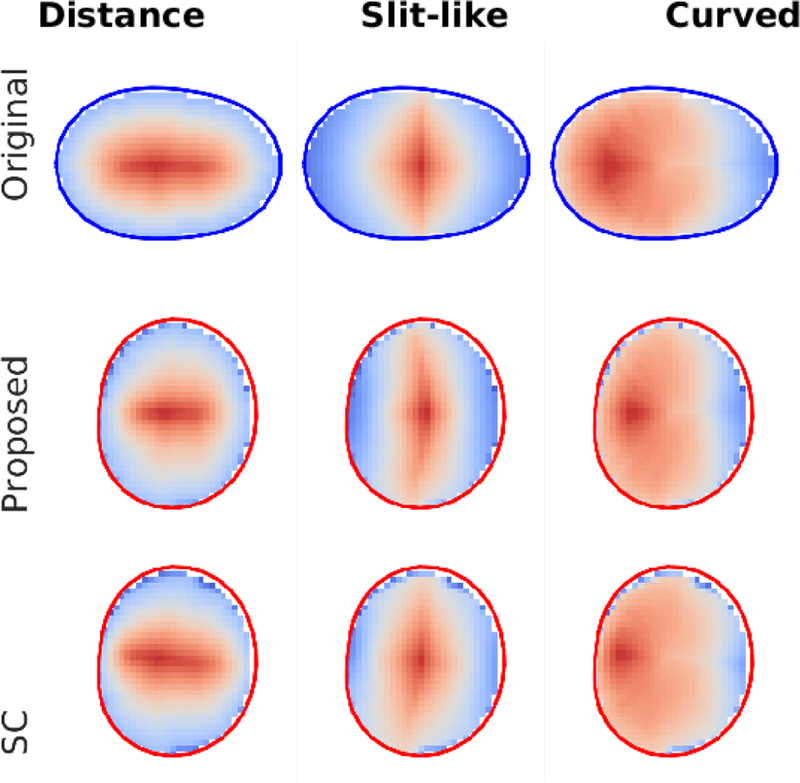
Mapped synthetic profiles for the case with highest dissimilarity between the SC method and the proposed method. The first row shows the original velocity from the synthetic imaging data, for three profile types. The second and third row show the velocities mapped to the model inlet (contoured in red). A notable difference is the angled profile in the slit-like profile (central column) which is observed in the SC method but not in the proposed method.

To have an intuitive understanding on the meaning of the differences between the SC mapping and the proposed method, Fig. 7 shows the profiles displayed the highest dissimilarity between the two methods.

### B. Results on In-vivo Data

In this section we show the results of the proposed method applied to two different datasets corresponding to a healthy volunteer and a cardiac patient. A more thorough description of the CFD results obtained using the of the proposed method on a larger number of patients can be found in [7], [13], [40].

Table II shows the distance between the velocity distributions before and after mapping, measured through the QC distance [39] between velocity histograms as described in Sec. IV. The columns show the results on patient data (velocity derived from MRI) and on data from a healthy volunteer (velocity derived from 3D CDI) obtained with the proposed method and the SC method.

**Fig. 8:**
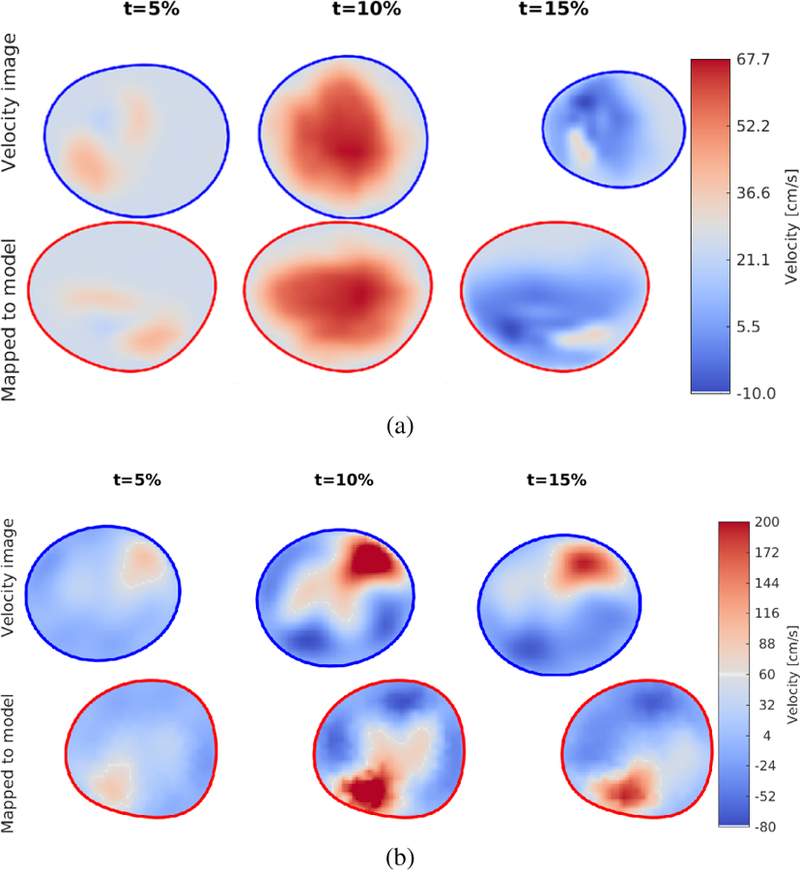
Velocity profile mapping addressing orientation and shape changes between the model and the velocity data. a) Profiles obtained from CDI images from a healthy volunteer. b) Profiles obtained from Flow MRI images from a cardiac patient

Fig. 8 shows the mapped velocity profile at *t* = 5% cycle duration (left column), *t* = 10% cycle duration (middle column) and *t* = 15% cycle duration (right column), for the healthy volunteer (Fig. 8a) and the patient with BAV and aortic stenosis (Fig. 8b). For each subfigure, the top row shows the input profile from the velocity imaging and the bottom row shows the velocity profile mapped to the model inlet. The profiles are coloured by velocity (note that scales are different for the volunteer and the patient since the stenotic valve forced a high velocity through the aortic inflow). Note that the profile from the healthy subject is centred within the inlet geometry and has a relatively wide plateau, while the BAV patient has a narrower, eccentric profile due to the diseased valve. Also note that while the shape of the mapped velocity profile is the same over time, the cross section in the imaging data varies. The profile rotation between the imaging data ant the model reflects the different orientation of the model and the velocity image, which was computed from the reference landmark.

**Fig. 9:**
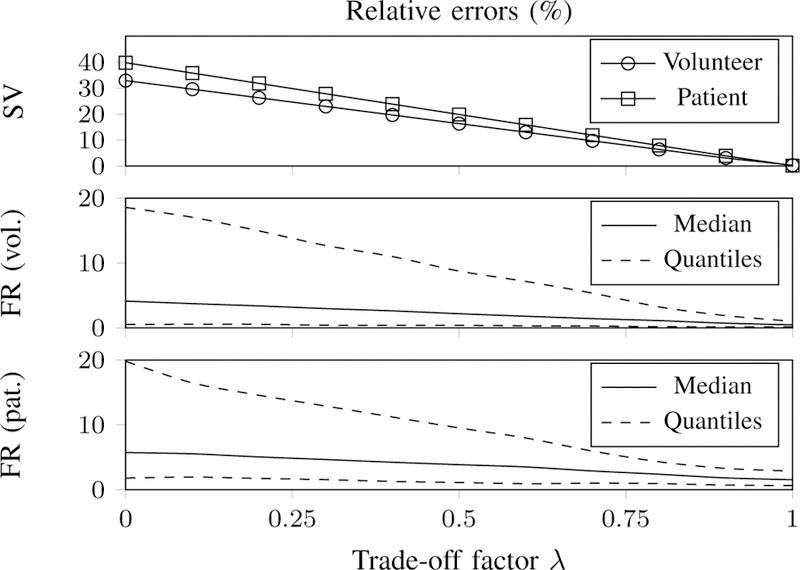
Quantitative analysis of the effect of the trade-off factor *λ*. (top) Relative absolute error in stroke volume (SV) for λ∈[0.1]. (middle) Relative absolute error in flow rate (FR), for the volunteer, showing the median and quantiles (25% and 75%) of the error distribution over the cardiac cycle. (bottom) Relative error in FR for the patient.

Fig. 9 (top) shows the relative errors in stroke volume (SV) for the volunteer and the patient. The middle and bottom panels of Fig. 9 show relative errors over the cardiac cycle (median and 25%–75% quantiles) in flow rate (FR), for the volunteer and the patient. Since the scaled velocity *v*_*f*_ (12) is linear with the interpolated velocity, it could be expected that the error in flow rate will decrease linearly when increasing *λ*. However it can be seen that for *λ >* 0.8 the error curves do not decrease linearly any more. This is due to interpolation errors which are averaged out when calculated global quantities integrated over the entire cycle. This can be seen in the plot at the top, where the error in SV decreases linearly with *λ*.

**Fig. 10:**
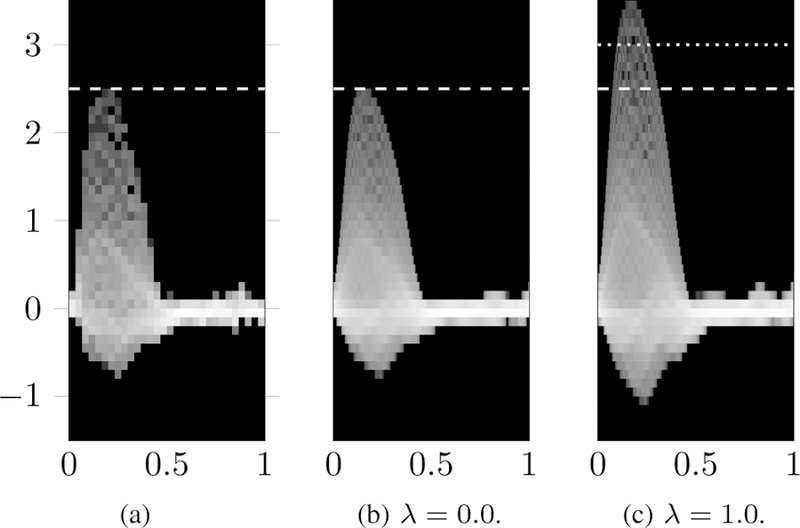
Blood velocity distribution (in m/s) of the mapped profile over time from a) imaging data, compared to the imaging data for different trade-off value *λ*, from b) matching the velocity distribution (*λ* = 0) to c) matching the flow rate (*λ* = 1).

Fig. 10 show an example of this effect for the patient data. Fig. 10a shows the histogram of through-plane velocities (along the vertical axis, in m/s) over time for the velocity imaging data.

Fig. 10b and 10c show the trace for the mapped model velocity profile over time, for *λ* = 0 and *λ* = 1 respectively. A white dashed line has been added at each figure to indicate the maximum systolic velocity. It can be seen that the maximum velocity increases linearly with *λ*, as expected, and more generally that the entire velocity distribution is linearly affected by the scaling.

**Fig. 11:**
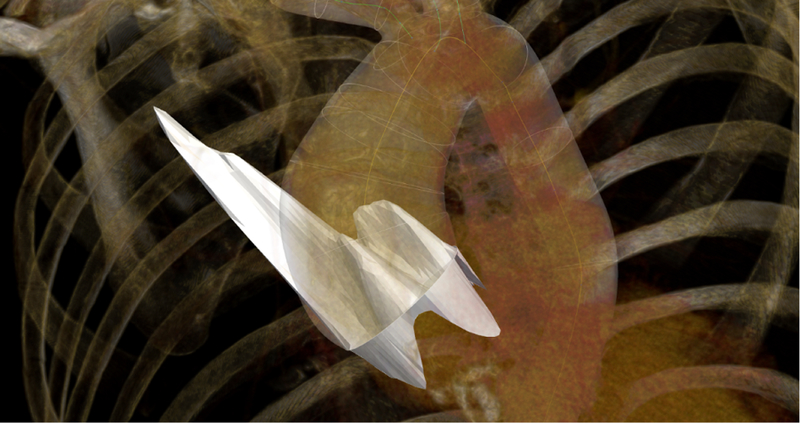
Visualisation of the profile mapping results in the CRIMSON softwareExample of visualization of the systolic profile prescribed on the inlet of the geometric model in CRIMSON [16]. b) Flow waveform illustrating the cyclic temporal interpolation GUI.

Fig. 11 renders the mapping of the PC-MRI velocity data to the inlet of the anatomical data of the BAV patient in the display panel of CRIMSON [16]. The visualization includes the finite element mesh of the aortic geometry, a volume render of the anatomy image data, and the 3D velocity profile(in white) imposed on the inlet face of the model(Fig. **??**). The original and interpolated flow waveforms and total cardiac output are also showncan be found in CRIMSON under the ‘Time interpolation settings’ panel at the bottom of the velocity mapping GUI(Fig. ??), alongside the cyclic time interpolation settings controlling the smoothness and sampling of the B-spline interpolator. Detailed instructions on how to perform the operations described on this paper can be found here: http://www.crimson.software/documentation.html.

## VI. Discussion

In this paper we proposed a method to map the velocity profile obtained from segmented velocity images (Flow MRI or colour Doppler images) onto a given face of the geometric vascular model for subject-specific CFD simulations of hemodynamics. The mapping consists of a series of rigid and non-rigid transformations that, combined, yield a dense, continuous and smooth deformation field that covers the inlet boundary and its surface.

The proposed method requires a segmentation of the face of interest in the velocity imaging data over the entire cardiac cycle, a corresponding point between the velocity and the anatomy data, and defining a factor *λ* between 0 and 1 which controls the trade-off between maintaining the velocity distribution or the flow rate through the mapping process. The method is fully automatic and sSince the mapping is continuous, the velocity profile can be mapped to any description of the geometry (e.g., discrete or analytical).

We have compared our proposed mapping method with the Schwarz-Christoffel (SC) mapping, which is widely used in related literature. Using synthetic data, our method produced similar results to the SC method, while running significantly faster.

In addition to testing in synthetic data, we have demonstrated the profile mapping on a healthy volunteer and a patient with bicuspid aortic valve (BAV), the commonest congenital cardiac defect, with a prevalence of 1–2%. Its morbidity and mortality amount to more than that of all other congenital cardiac conditions combined [14]. It is commonly associated with aneurysms of the thoracic aorta [15], and the hemodynamic link between BAV morphology and aneurysm formation is the current topic of intense research. Fig. 8 shows that velocity profiles can be very different to idealised profiles. Therefore, image-based CFD analysis constitutes a non-invasive tool to investigate this hemodynamic relationship between valve morphology and aneurysm formation. The ability to use patient-specific velocity profiles has the potential to improve CFD simulations, and lend further insight into this common disease process.

In the approach presented here, we have assumed that the geometry of the vascular model is described by a single temporal phase. This situation is typically encountered in rigid wall or in linearised fluid-structure interaction simulations, such as those performed using the coupled-momentum method [2]. Given that the velocity data must be segmented for all its temporal phases, this will in general lead to a difference between the effective surface area of the face of the geometric model and the corresponding areas in the velocity image data. Therefore, the user must specify the value of a trade-off parameter *λ* depending on the target application. To the best of our knowledge, most previous work has chosen to maintain flow rate (*λ* = 1). However, applications where velocity-dependent metrics are to be derived from the simulation results, such as wall shear stress, may benefit from *λ* = 0, or intermediate values of *λ*.

The work presented here could be generalized for situations in which multiple phases are available to describe the anatomical images. In general, the number of phases would be the same as those of the velocity images. In this scenario, anatomical landmarks must would have to be specified for each phase, and rotational alignments between velocity and model contours performed. In this case, the time series of contours for the face of interest would be identical for the velocity and anatomical images, and thus the trade-off scaling parameter would be set to zero. This situation would therefore define boundary conditions for velocity and vessel motion in large deformation fluid-structure interaction problems [1]. Moreover, because both the frame-wise mapping (2D) and the temporal smoothing (2D+t) are carried out using B-splines, both steps could be merged into a 2D × 2D+t (5D) B-spline formulation. Implications of the merge in terms of efficiency, accuracy and need for regularization are out of the scope of this paper.

We have implemented the proposed mapping method in a publicly available hemodynamic simulation software (CRIMSON) to enable wide penetration of the method and make it possible for researchers and clinicians to use patient-specific velocity boundary conditions in their hemodynamic simulations.

## VII. Limitations

In this paper, we have assumed that the blood velocity is parallel to the vessel wall on the plane defining the velocity data. Several studies [6], [23] have shown that the effect of this simplification is small and does not affect the outcome of the simulations significantly. It can however affect the simulated flow near the inlet; for applications targeting regions in the vicinity of the valves this limitation should be addressed. In this region also, and particularly for patients with abnormal valves, the in-plane component of the flow may play an important role [12] and should be considered.

## VIII. Conclusion

We have presented a novel method to map patient-specific, time-resolved velocity profiles from imaging data (colour Doppler or flow MR) to a boundary face of a geometric vascular model. Our method enables to maintain either the flow rate or the velocity distribution through the mapping process and addresses changes in orientation, shape and size between the velocity imaging data and the anatomical model. The resulting profiles are smooth, temporally cyclic and time-resolved.

The proposed method allows the inclusion of patient-specific inflow profiles into CFD workflows, which has the potential of rendering more accurate and realistic simulations. This is particularly the case when abnormal velocity profiles are an important characteristic of the disease under study, as in the case of aortic valve disease. Lastly, we have made the method accessible to the wider community through the open source hemodynamic simulation software CRIMSON [16].

## Figures and Tables

**Fig. 12:**
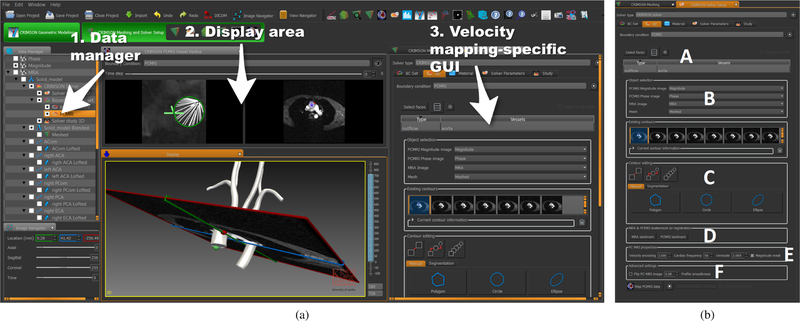
CRIMSON GUI overview. a) 1. Data manager with all the loaded objects, 2. Display area used for data visualisation, and 3. Activity-specific GUI controls are located on the right side. In this example the selected activity is velocity profile mapping. b) Detailed view of the CRIMSON velocity profile mapping GUI, which includes: A) Model face selection. B) Specification of velocity and anatomy images and geometric model. C) Vessel lumen segmentation in the velocity images. D) Positioning of landmarks in both model and velocity images. E) Additional information related to the velocity image in the context of PC-MRI (such as velocity encoding). F) Advanced settings.

**Fig. 13:**
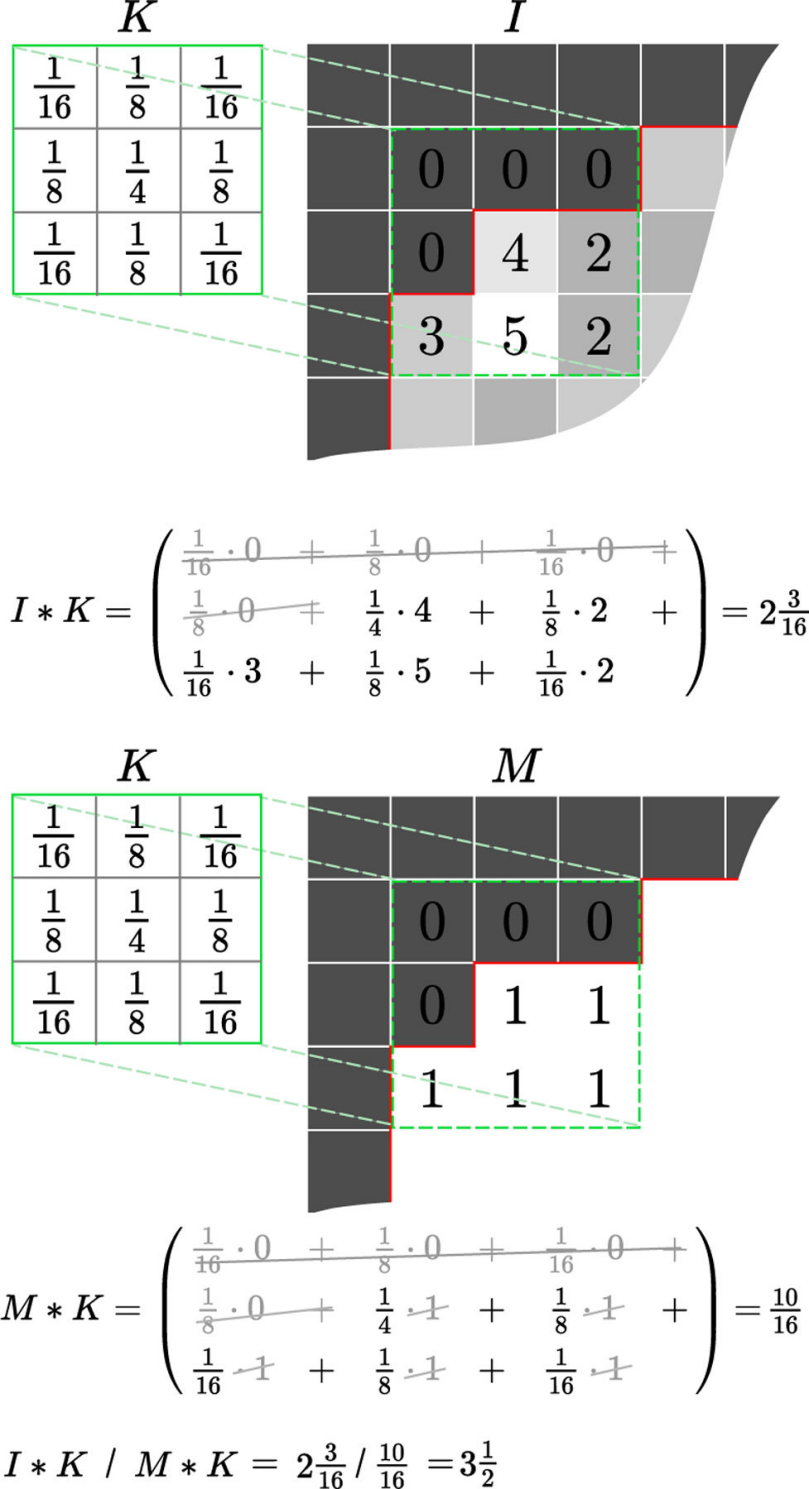
Gaussian blurring with arbitrary boundaries. Convolution of an image *I* with a 3×3 Gaussian kernel *K*, where the red line depicts the boundary of our domain mask *M*. By zeroing all values outside of our domain they do not contribute to the weighted average *I* ∗*K*. In this case we average five values only. The weighted average $\left(2\frac{3}{16}\right)$ is lower than the values that contributed to it as the weights used do not sum to one. Convolving the binary domain mask with the kernel, *M* ∗ *K*, yields the total weight $\left(\frac{10}{16}\right)$, which is used to normalise the final result, (I∗K)/(M∗K), giving a better average $\left(3\frac{1}{2}\right)$.

**TABLE I:** QC [39] DISTANCE IN VELOCITY DISTRIBUTION AFTER MAPPING FOR THREE SYNTHETIC PROFILE TYPES

	Distance	Slit-like	Curved
Proposed	3.9 ± 3.6	3.9 ± 3.7	3.9 ± 3.9
SC^2^	3.6 ± 3.4	4.1 ± 3.5	4.0 ± 3.7

1 Average ± standard deviation

2 Schwarz-Christoffel (SC) method

**TABLE II:** QC [39] DISTANCE IN VELOCITY DISTRIBUTION AFTER MAPPING FOR A PATIENT AND A HEALTHY VOLUNTEER.

	Flow MRI (patient)	Colour Doppler (volunteer)
Proposed	2.8 ± 1.4	8.5 ± 13.8
SC^2^	2.9 ± 1.4	9.0 ± 13.3

1 Average ± standard deviation

2 Schwarz-Christoffel (SC) method

## APPENDIX A

### A Freely Available Implementation of the Method as part of CRIMSON

CRIMSON [16], the CardiovasculaR Integrated Modelling and SimulatiON environment (www.crimson.software), is a software package that provides a complete pipeline for image-based vascular segmentation, modelling, large-scale blood flow simulation and analysis. The software is designed to be both intuitive for clinical use and easily extensible for advanced research users. Based on a series of well-established open source libraries, including MITK [32], vmtk [33], Verdandi [34] and QSapecNG [35], among others, and with a global user-base, CRIMSON is an ideal platform for disseminating the methods described in the present article and achieve widespread penetration, accessibility and reproducibility.

A general view of the CRIMSON graphical user interface (GUI) is shown in Fig. 12a. The GUI contains three main areas: 1. Data manager with intermediate results of different operations (vessel tree, vessel model, mesh, solver settings, etc.), 2. Display area, and 3. Module-specific GUI, which in this case shows the velocity profile mapping controls detailed in Fig. 12b.

The velocity profile mapping implementation in CRIMSON requires a blood velocity image and an anatomical image from which the vascular model will be obtained by first segmenting the lumen boundary from the anatomical image and then meshing the segmented volume. Alternatively, vascular models created with other packages can also be imported into CRIMSON.

The profile mapping tool provides a visualisation panel (central area in Fig. 12a), whose top part is divided into two smaller subpanels. The left subpanel shows a 2D slice of the anatomy image at the inlet face onto which we wish to map the velocity profile. The right subpanel shows one time-resolved slice of the velocity image. This subpanel enables the user to segment the vessel wall in the velocity image at any desired phase of the cardiac cycle using a time step slider, and to place landmarks (see Figs. 1 and 2) in both the anatomical and velocity images. It should be noted that in the current paradigm (for which a single anatomical image is considered), while the vessel wall segmentation needs to be performed for each phase of the velocity image, the anatomical landmark needs to be defined only for the first phase. The bottom part of the display area shows a 3D view of the vascular model, an anatomical landmark (green sphere) and the spatially aligned 2D velocity image relative to the 3D anatomical image. For easier identification of the reference points, the landmark is rendered as a green cross (2D) and a sphere (3D) in the anatomical and velocity images, respectively.

The velocity profile mapping control panel is shown in detail in Fig. 12b. This panel allows the user to set all required inputs for the velocity profile mapping: (A) the geometric model face onto which the velocity profile will be mapped, (B) the velocity and the anatomy images along with the geometric model, (C) the controls for manual and semi-automatic segmentation of vessel lumen in the velocity images, (D) an interface for the placement of the corresponding landmarks, (E) acquisition- and manufacturer-dependent velocity settings (in the context of PC-MRI [36]), such as velocity encoding, cardiac frequency and velocity scale, and (F) advanced settings including the possibility to flip the orientation of the velocity image and the value of variance for the Gaussian smoothing filter. The Gaussian smoothing filter may be applied to the masked velocity image before the mapping described in Sec. III-A to make the velocity profile smoother. Besides the Gaussian filter the smoothing also implements the suppression of undesirable edge-effects typically found on masked images (Appendix B).

## APPENDIX B

### Mass-preserving Convolution near the Edge of Arbitrarily Shaped Image Domains

Linear filtering operations on images, such as Gaussian blurring, can be efficiently implemented through 2D convolutions. Convolution is an operation where each pixel becomes a weighted average of itself and its neighbours within a rectangular neighbourhood. This process is typically visualised as “sliding” a kernel weighting matrix *K* over an image *I* and computing a sum of products within a window. The weights in the kernel matrix *K* are usually normalised so that they sum to one, ensuring the average intensity is preserved and the image does not become darker or lighter.

Often times, as is the case in this paper, data is confined in an arbitrarily shaped domain within the image (e.g. inside the contour of a vessel cross section). Since the values outside the segmented area are zero, a standard convolution would artificially push values near the boundary towards zero. In this paper we propose a simple renormalisation scheme which is easily implemented via two convolutions, as exemplified in Fig. 13.

Setting all values to zero outside of our domain mask *M*, and computing the convolution *I* ∗ *K* is equivalent to computing a weighted average of values, restricted to our domain, albeit where our weights no longer sum to one (leading to darker - i.e. closer to zero - boundary values). We can correct this for each pixel by normalising by the total weight of the pixels inside the domain and under the current location of the kernel, and ignoring the weight of those which lie outside the domain. This total weight for each pixel may be computed by convolving a binary domain mask *M* with the kernel, *M* ∗ *K*. Thus the normalised convolution is (*I* ∗ *K*)*/*(*M* ∗ *K*).
